# A Mobile Instant Messaging–Delivered Psychoeducational Intervention for Cancer Caregivers: A Randomized Clinical Trial

**DOI:** 10.1001/jamanetworkopen.2023.56522

**Published:** 2024-02-22

**Authors:** Qinqin Cheng, Marques Shek Nam Ng, Kai Chow Choi, Yongyi Chen, Gaoming Liu, Winnie Kwok Wei So

**Affiliations:** 1 Hunan Cancer Hospital, Changsha, Hunan, China; 2 Nethersole School of Nursing, Faculty of Medicine, Chinese University of Hong Kong, Hong Kong, China

## Abstract

**Importance:**

Psychoeducational interventions (PEIs), which provide both information and emotional and psychosocial support, may address the unmet needs of the caregivers of adolescent and young adult patients with cancer.

**Objective:**

To explore the effects of an instant messaging–delivered PEI on anxiety, depression, quality of life (QOL), and coping and determine whether the intervention reduces caregivers’ unmet needs.

**Design, Setting, and Participants:**

This randomized clinical trial using an intention-to-treat protocol was conducted from April 1 to September 14, 2022, in a tertiary cancer hospital in China and included caregivers of patients diagnosed with cancer at age 15 to 39 years recruited using convenience sampling.

**Intervention:**

Caregivers were allocated 1:1 using a randomized block scheme to the intervention or control group. The intervention group received a 5-week PEI and usual care, whereas the control group received only usual care. The PEI was delivered through articles and calls through an instant messaging application.

**Main Outcomes and Measures:**

The primary outcomes were changes in the levels of caregivers’ anxiety and depression, measured using the 7-Item Generalized Anxiety Disorder Scale and the Patient Health Questionnaire 9, respectively. The secondary outcomes were changes in the levels of caregivers’ QOL, coping, and unmet needs.

**Results:**

Of the 160 participants, 92 (57.5%) were male; mean (SD) age was 40.27 (8.33) years. Compared with the control group, the intervention group had significantly greater reduction in severity of anxiety (B = −3.231; 95% CI, −4.746 to −1.716; *P* < .001) and depression (B = −3.253; 95% CI, −5.052 to −1.454; *P* < .001), smaller reduction in QOL (B = 13.574; 95% CI, 0.488-26.661; *P* = .04), and greater reduction in unmet needs (B = −12.136; 95% CI, −18.307 to −5.965; *P* < .001) after the intervention. Twelve weeks after baseline, the intervention group demonstrated a significantly greater reduction only in severity of anxiety (B = −1.890; 95% CI, −3.382 to −0.397; *P* = .01).

**Conclusions and Relevance:**

In this randomized clinical trial of a mobile instant messaging–delivered PEI, caregivers’ unmet needs, anxiety, and depression decreased significantly and QOL declined at a significantly slower rate immediately after the intervention in the intervention group compared with the control group. A sustained effect on anxiety was observed 12 weeks after baseline.

**Trial Registration:**

Chinese Clinical Trial Registry identifier: ChiCTR2200055951

## Key Points

**Question:**

Can a mobile instant messaging–delivered psychoeducational intervention (PEI) relieve anxiety and depression for caregivers of adolescent and young adult patients with cancer?

**Findings:**

In this randomized clinical trial involving 160 caregivers of patients diagnosed with cancer at age 15 to 39 years, a mobile instant messaging–delivered PEI significantly reduced caregivers’ anxiety and depression immediately after the intervention. A sustained effect on anxiety was observed 12 weeks after baseline.

**Meaning:**

The findings suggest that the mobile instant messaging–delivered PEI could be routinely used to reduce anxiety and depression among caregivers of adolescent and young adult clinical oncology patients.

## Teaser

This randomized clinical trial investigates whether a psychoeducational intervention delivered by instant messaging reduces anxiety and depression and improves coping and quality of life for caregivers of adolescents and young adults with cancer.

## Introduction

Adolescent and young adult (AYA) patients with cancer, diagnosed at 15 to 39 years of age,^1^ rely primarily on their parents or spouses as main caregivers.^2^ These caregivers, often considered as secondary patients, experience significant stress and long-term responsibilities.^3,4^ They face unique challenges distinct from those of caregivers of children or older adults given the various unique medical and psychosocial concerns of AYA patients with cancer.^5,6^ When experiencing such stress, caregivers often have inadequate coping strategies^7^ and many unmet needs, particularly informational and emotional.^8,9^ In addition to common caregiver needs, they have special needs related to unique psychosocial concerns of AYA patients with cancer and more pronounced worries about cancer recurrence and secondary cancer.^10^ Consequently, they report an impaired quality of life (QOL),^11^ with one-third experiencing moderately to severely elevated anxiety and depressive symptoms.^12,13^

There is a limited number of interventions specifically designed for caregivers of AYA patients with cancer. To date, 1 coping support intervention^14^ and 1 self-care and communication intervention have been reported.^15^ However, these interventions focused on enhancing the functional aspects of coping or communication and did not address all unmet needs of caregivers. Other interventions for caregivers of AYA patients with cancer are therefore needed to address their unmet needs.

Psychoeducational interventions (PEIs) are commonly used to address the needs and challenges of caregivers.^16^ Considering the common unmet needs of caregivers of AYA patients with cancer,^8,9^ a PEI that provides information and addresses their emotional and psychosocial needs may be a suitable intervention.^16^ Previous studies have shown that PEIs are effective at reducing unmet needs, relieving psychological stress, and enhancing QOL in caregivers of adult patients with cancer.^17,18,19,20,21^ However, the content of these PEIs may not be directly applicable to caregivers of AYA patients with cancer.^22^ Therefore, guided by stress and coping theory,^23^ we developed a PEI to address this gap. The theoretical framework and the rationale for this intervention have been described previously.^24^

After developing the PEI, we conducted a pilot study^24^ to examine its feasibility and acceptability. Considering the caregivers’ varying schedules and the patients’ treatment cycles, the PEI was delivered using a mobile instant messaging application; these have been increasingly used for caregiver interventions as they allow flexibility regarding time and location.^25,26,27^ The pilot study indicated that this mobile instant messaging–delivered PEI was feasible for and acceptable to caregivers of AYA patients with cancer.^24^ We then conducted a full-scale randomized clinical trial to explore the effects of the PEI on anxiety, depression, QOL, and coping and to determine whether it reduced the unmet needs of caregivers of AYA patients with cancer.

## Methods

### Study Design

This prospective, 2-arm, parallel-group randomized clinical trial was conducted from April 1 to September 14, 2022 (ChiCTR2200055951). The trial protocol is provided in Supplement 1. Ethical approval for this study was obtained from the Joint Chinese University of Hong Kong–New Territories East Cluster Clinical Research Ethics Committee and the Hunan Cancer Hospital. Written informed consent was provided by the participants. The study followed the Consolidated Standards of Reporting Trials (CONSORT) reporting guideline.

### Setting and Participants

Caregivers of patients diagnosed with cancer at age 15 to 39 years were recruited from the inpatient wards of a tertiary cancer hospital in Changsha, Hunan Province, China, using convenience sampling. Caregivers were eligible if they (1) were primarily caring for an AYA patient with cancer in the treatment phase, (2) cared for the patient after discharge, (3) were aged at least 18 years, (4) had at least 1 unmet need of a high level according to the Support Person’s Unmet Needs Survey–Short Form,^28,29^ (5) understood the research procedures and could read and communicate in Chinese, (6) could be reached via a mobile instant messaging app, and (7) provided informed consent to participate in the study. Caregivers were excluded if they (1) were being paid, (2) cared for an AYA patient with cancer receiving hospice care, (3) had a mental health condition or cognitive impairment resulting in inability to participate in the intervention, or (4) were participating in another research interventional program.

The sample size of the study was estimated based on the primary outcomes (changes in the levels of anxiety and depression) using G*Power, version 3.1.9.7 (University of Dusseldorf).^30^ Given that to our knowledge, no similar study has been conducted in this age group, the effect sizes of the primary outcomes of previous studies of PEIs for caregivers of adult patients with cancer were considered. The pooled effect sizes for anxiety and depression in previous studies were 0.50 and 0.54, respectively.^31^ It was estimated that a sample size of 64 participants in each arm of a 2-parallel-arm trial was required to detect an effect size of at least 0.50 in the change scores of the primary outcomes after the intervention, with 80% power at a 2-tailed 5% significance level. Considering a potential attrition rate of 20%, the required sample size was 160 participants, with 80 participants per arm.

### Randomization, Allocation, and Masking

The participants were randomly allocated to the intervention or control group at a 1:1 ratio using a randomized block design with varying block sizes of 4 or 6 to maintain a good balance of participants and enhance allocation concealment throughout the recruitment period. A research assistant not involved in participant recruitment or data collection generated a random sequence of 160 using an online randomizer.^32^ The allocation sequence was concealed using sealed opaque envelopes and preserved by the research assistant, who was not involved in other processes of this study. Once informed consent was obtained, the envelopes were sequentially opened by the same research assistant to disclose the allocation results. Considering the nature of PEIs, blinding the intervenor and participants was not feasible. Moreover, as all of the study’s outcomes were self-reported, the outcome assessors (ie, the participants themselves) also were not blinded.

### Control Group

Participants in the control group received usual care, including routine inpatient and discharge education. The hospital’s telephone helpline number was also provided.

### Intervention Group

In addition to usual care, participants in the intervention group received the PEI, which was delivered by the principal investigator (PI) (Q.C.). The details of the development and delivery procedure of the intervention were described in the previous pilot study.^24^ In brief, according to stress and coping theory, caregivers’ unmet needs and coping strategies were 2 modifiable factors that were targeted to improve their health outcomes.^23^ Therefore, the contents of the PEI were developed based on 2 coping functions (problem-based and emotion-based coping), with consideration of the caregivers’ unmet needs regarding information, finances, health care access and continuity, worries about the future, and personal and emotional needs. Specific psychosocial needs related to AYA patients with cancer, such as addressing fertility issues,^5^ were also included in the intervention. Details about how the content was developed based on corresponding coping functions and needs (Table 1) have been reported previously.^24^

**Table 1.** Mobile Instant Messaging–Delivered PEI for Caregivers of AYA Patients With Cancer

Session	Coping functions	Needs	Content	Duration, min
1	Problem-based	Information, financial, and health care access and continuity	Provide information on cancer, treatments, and adverse effects Provide information on medical insurance and financial assistance program Teach caregivers how to obtain health care services Teach caregivers how to communicate with health care professionals effectively	30-45
2	Problem-based	Information	Provide information on AYA populations and their fertility issues Educate caregivers on how to help manage patients’ illness at home Educate caregivers on how to communicate and talk about the illness with patients	30-45
3	Emotion-based	Personal and emotional	Teach caregivers how to care for themselves Encourage caregivers to share their stress and emotions Teach caregivers how to manage their stress Teach caregivers how to regulate negative emotions	30-45
4	Emotion-based	Worries about the future	Discuss caregivers’ uncertainties or worries Teach caregivers how to cope with uncertainty Encourage optimistic thinking	30-45
5	NA	Other	Discuss caregivers’ other needs	10-20

Abbreviations: AYA, adolescent and young adult (diagnosed at age 15-39 years); NA, not applicable; PEI, psychoeducational intervention.

The PI delivered the intervention via a popular mobile instant messaging app in China. The first session was initiated within 1 week of caregiver recruitment followed by 4 consecutive weekly sessions. During the intervention, the educational content, presented in articles, was sent to the caregivers weekly through instant messaging. The PI then used a teach-back strategy to confirm the participants’ understanding and clarify any misunderstandings. The PI also discussed the caregivers’ problems or concerns and possible coping strategies individually in each session through calls on the instant messaging app.

The intervention was delivered according to an intervenor manual to ensure consistency. Appealing articles and reminder messages were used to enhance adherence. To protect the participants’ security and privacy, only research-related information was discussed, and their personal information was not disclosed to others.

### Outcomes and Measurements

The eAppendix in Supplement 2 provides detailed descriptions, including the psychometric properties, of each measurement. The primary end points were changes in the levels of caregivers’ anxiety and depression at 5 weeks (T1) and 12 weeks (T2) after baseline with respect to baseline levels (T0). Anxiety was measured using the 7-Item Generalized Anxiety Disorder Scale.^33,34^ Depression was measured using the Patient Health Questionnaire 9.^35,36^ The secondary end points were changes in the levels of caregivers’ QOL, coping, and unmet needs at T1 and T2 relative to T0 as assessed using the Quality of Life Scale–Family Version,^37,38^ the Brief Coping Orientations to Problems Experienced Scale,^39,40^ and the Support Person’s Unmet Needs Survey–Short Form,^28,29^ respectively. In addition, participants’ sociodemographic and clinical characteristics were collected.

### Data Collection Procedure

Before baseline data collection, the unmet needs of potential participants were assessed to determine their eligibility. The outcomes except for unmet needs were assessed at T0, T1, and T2. Participants’ unmet needs were reassessed immediately after the intervention (T1) to determine whether their unmet needs were satisfied. Baseline data were collected on site by the PI and research assistants using paper questionnaires, while follow-up data were collected using electronic questionnaires,^41^ which were sent by the PI.

### Statistical Analysis

All data were analyzed using SPSS, version 26.0 (IBM Corp). Continuous data were evaluated for normality by assessing skewness and kurtosis and by a visual inspection of Q-Q plots.^42,43^ Potential outliers were identified using a criterion of more than 3 absolute deviations from the median.^44^ No continuous variable was found to violate the normal distribution, and no outliers were detected. Continuous variables are presented as means (SDs), while categorical variables are expressed as numbers and percentages. A generalized estimating equations (GEE) model was used to compare the changes in each of the outcome scores at T1 and T2 with respect to T0 between the intervention and control groups.^45^ Two group-by-time interaction terms (group × T1 and group × T2) were included in the model to assess the changes. The intention-to-treat principle was followed as all of the randomized participants had complete baseline data and were included in the GEE analysis.^46^ The built-in quasi-likelihood method for parameter estimation in SPSS was used to handle missing data in the GEE analysis, which can produce unbiased estimates even in the presence of missing data provided that the data are missing completely at random.^47^ As the aim of the study was not to confirm the effectiveness of the intervention but to explore its effects on the study outcomes, multiplicity adjustment was not required. Cohen *d* values were calculated to estimate the effect sizes of the outcome variables showing statistical significance.^48^ All statistical tests were 2-tailed, with the level of significance set at *P* < .05.

## Results

A total of 322 caregivers of AYA patients with cancer were screened for eligibility from April to June 2022, and 160 eligible caregivers were enrolled, including 68 females (42.5%) and 92 males (57.5%); mean (SD) age was 40.27 (8.33) years (Figure). The sociodemographic and clinical characteristics of the participants and their care recipients are displayed in Table 2. Follow-up was completed on September 14, 2022. The overall attrition rate was 20.0% 12 weeks after baseline, with rates of 17.5% and 22.5% for the intervention and control groups, respectively.

**Figure.**
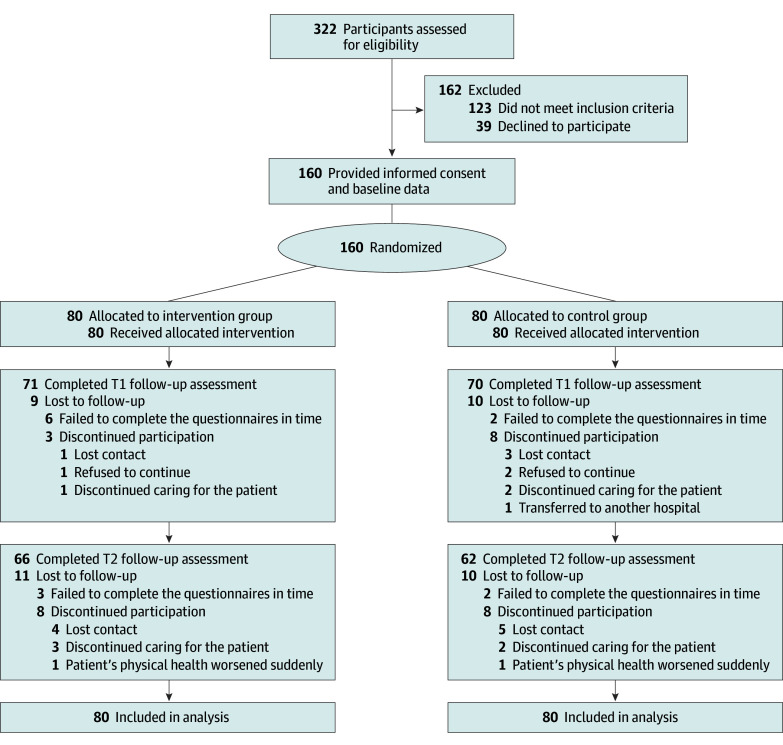
CONSORT Diagram of Participant Flow Through the Intervention T1 indicates 5 weeks after baseline; T2, 12 weeks after baseline.

**Table 2.** Sociodemographic and Clinical Characteristics of Caregiver Participants and AYA Patients With Cancer

Variable	Individuals^a^		
	Total (N = 160)	Intervention (n = 80)	Control (n = 80)
**Caregivers**			
Age, mean (SD), y	40.27 (8.33)	40.69 (8.72)	39.85 (7.95)
Sex			
Female	68 (42.5)	33 (41.3)	35 (43.8)
Male	92 (57.5)	47 (58.8)	45 (56.3)
Educational level			
Primary school or below	9 (5.6)	5 (6.3)	4 (5.0)
Junior high school	47 (29.4)	25 (31.3)	22 (27.5)
Senior high school	45 (28.1)	23 (28.7)	22 (27.5)
College or above	59 (36.9)	27 (33.8)	32 (40.0)
Marital status			
Married	153 (95.6)	78 (97.5)	75 (93.8)
Unmarried, widowed, or divorced	7 (4.4)	2 (2.5)	5 (6.3)
Place of residence			
Urban	66 (41.3)	33 (41.3)	33 (41.3)
Rural	94 (58.8)	47 (58.8)	47 (58.8)
Employment status			
Employed	102 (63.7)	52 (65.0)	50 (62.5)
Not employed	58 (36.3)	28 (35.0)	30 (37.5)
Monthly family income per capita, CNY			
≤1000	17 (10.6)	9 (11.3)	8 (10.0)
1001-3000	41 (25.6)	20 (25.0)	21 (26.3)
3001-5000	44 (27.5)	22 (27.5)	22 (27.5)
5001-8000	40 (25.0)	20 (25.0)	20 (25.0)
≥8001	18 (11.3)	9 (11.3)	9 (11.3)
Relationship to the patient			
Spouse	104 (65.0)	53 (66.3)	51 (63.7)
Parent	38 (23.8)	20 (25.0)	18 (22.5)
Sibling	18 (11.3)	7 (8.8)	11 (13.8)
**AYA patients with cancer**			
Age, mean (SD), y	32.90 (6.62)	33.05 (6.29)	32.75 (6.97)
Sex			
Female	115 (71.9)	60 (75.0)	55 (68.8)
Male	45 (28.1)	20 (25.0)	25 (31.3)
Medical insurance			
No	8 (5.0)	5 (6.3)	3 (3.8)
Yes	152 (95.0)	75 (93.8)	77 (96.3)
Type of cancer			
Breast	49 (30.6)	23 (28.7)	26 (32.5)
Gynecological	43 (26.9)	24 (30.0)	19 (23.8)
Head and neck	20 (12.5)	9 (11.3)	11 (13.8)
Lymphoma	15 (9.4)	6 (7.5)	9 (11.3)
Gastrointestinal and liver	13 (8.1)	7 (8.8)	6 (7.5)
Bone	12 (7.5)	4 (5.0)	8 (10.0)
Other^b^	8 (5.0)	7 (8.8)	1 (1.3)
Age when diagnosed, mean (SD), y	32.49 (6.68)	32.69 (6.41)	32.29 (6.98)
Cancer stage			
I	32 (20.0)	18 (22.5)	14 (17.5)
II	27 (16.9)	14 (17.5)	13 (16.3)
III	50 (31.3)	25 (31.3)	25 (31.3)
IV	36 (22.5)	13 (16.3)	23 (28.7)
Unclear	15 (9.4)	10 (12.5)	5 (6.3)
Current cancer treatment			
Surgery	45 (28.1)	22 (27.5)	23 (28.7)
Chemotherapy	115 (71.9)	58 (72.5)	57 (71.3)

Abbreviations: AYA, adolescent and young adult (diagnosed at age 15-39 years); CNY, Chinese yuan.

^a^ Data are presented as number (percentage) of participants unless otherwise indicated.

^b^ Brain cancer, lung cancer, kidney cancer, bladder cancer, and sarcoma.

The main reasons for attrition were loss of contact, refusal to continue, discontinuance of caring for the patient, transfer to another hospital, or a sudden deterioration in the patient’s physical health. Furthermore, the baseline characteristics and reasons for dropping out were similar between the participants who completed the study and those who dropped out (eTable 1 in Supplement 2), which supports the conclusion that data were missing completely at random. Table 3 summarizes the primary and secondary outcomes and the effect sizes.

**Table 3.** Generalized Estimating Equations Models for the Comparison of Each Outcome Across Time Between the Intervention and Control Groups

Outcome variable, measure	Score, mean (SD)		Group coefficient		Time coefficient		Group × time coefficient		Cohen *d*
	Intervention	Control	B (95% CI)	*P* value	B (95% CI)	*P* value	B (95% CI)	*P* value	
**Primary outcomes**									
Anxiety, GAD-7^a^									
T0	9.69 (5.78)	8.96 (5.24)	0.725 (−0.973 to 2.423)	.40	[Reference]	NA	[Reference]	NA	NA
T1	7.13 (4.48)	9.26 (5.15)	NA		0.632 (−0.329 to 1.593)	.20	−3.231 (−4.746 to −1.716)	<.001	0.71
T2	7.74 (5.06)	8.56 (5.09)	NA		−0.129 (−1.134 to 0.875)	.80	−1.890 (−3.382 to −0.397)	.01	0.47
Depression, PHQ-9^b^									
T0	8.05 (6.26)	7.40 (5.69)	0.650 (−1.192 to 2.492)	.49	[Reference]	NA	[Reference]	NA	NA
T1	6.58 (4.88)	8.74 (6.40)	NA		1.685 (0.491 to 2.879)	.006	−3.253 (−5.052 to −1.454)	<.001	0.59
T2	7.12 (4.89)	7.53 (5.67)	NA		0.539 (−0.708 to 1.786)	.40	−1.476 (−3.259 to 0.308)	.11	NA
**Secondary outcomes**									
Quality of life, QOL-Scale FAM^c^									
T0	178.65 (59.44)	170.04 (47.73)	8.613 (−7.989 to 25.214)	.31	[Reference]	NA	[Reference]	NA	NA
T1	174.07 (58.73)	154.89 (46.32)	NA		−17.081 (−25.976 to −8.186)	<.001	13.574 (0.488 to 26.661)	.04	0.31
T2	168.71 (60.62)	156.32 (52.49)	NA		−15.433 (−24.572 to −6.294)	.001	6.236 (−7.662 to 20.134)	.38	NA
Coping, Brief-COPE									
Problem-focused^d^									
T0	14.98 (3.59)	15.60 (2.58)	−0.625 (−1.589 to 0.339)	.20	[Reference]	NA	[Reference]	NA	NA
T1	15.24 (3.04)	14.83 (3.12)	NA		−0.709 (−1.467 to 0.048)	.07	0.934 (−0.181 to 2.050)	.10	NA
T2	14.47 (2.98)	14.94 (2.80)	NA		−0.655 (−1.349 to 0.039)	.06	0.149 (−0.880 to 1.178)	.78	NA
Emotion-focused^e^									
T0	21.78 (4.38)	22.30 (3.92)	−0.525 (−1.804 to 0.754)	.42	[Reference]	NA	[Reference]	NA	NA
T1	22.15 (3.86)	22.06 (3.76)	NA		−0.146 (−1.011 to 0.719)	.74	0.473 (−0.799 to 1.745)	.47	NA
T2	21.21 (3.34)	20.73 (3.08)	NA		−1.494 (−2.455 to −0.533)	.002	0.973 (−0.434 to 2.380)	.18	NA
Dysfunctional^f^									
T0	22.73 (5.11)	23.19 (3.98)	−0.462 (−1.873 to 0.948)	.52	[Reference]	NA	[Reference]	NA	NA
T1	22.61 (4.65)	23.27 (4.43)	NA		0.299 (−0.553 to 1.150)	.49	−0.356 (−1.613 to 0.900)	.58	NA
T2	22.11 (4.33)	22.77 (4.05)	NA		−0.134 (−1.102 to 0.833)	.79	−0.560 (−1.863 to 0.744)	.40	NA
Unmet needs, SPUNS-SF^g^									
T0	49.86 (17.11)	49.40 (18.10)	0.463 (−4.961 to 5.886)	.87	[Reference]	NA	[Reference]	NA	NA
T1	25.73 (13.96)	36.81 (16.44)	NA		−12.397 (−16.763 to −8.031)	<.001	−12.136 (−18.307 to −5.965)	<.001	0.69

Abbreviations: Brief-COPE, Brief Coping Orientations to Problems Experienced Scale; GAD-7, 7-Item Generalized Anxiety Disorder Scale; NA, not applicable; PHQ-9, Patient Health Questionnaire 9; QOL-Scale FAM, Quality of Life Scale–Family Version; SPUNS-SF, Support Person’s Unmet Needs Survey–Short Form; T0, baseline; T1, 5 weeks after baseline; T2, 12 weeks after baseline.

^a^ Ranges from 0 to 21, with higher scores indicating worse anxiety.

^b^ Ranges from 0 to 27, with higher scores indicating worse depression.

^c^ Ranges from 0 to 350, with higher scores indicating better quality of life.

^d^ Ranges from 6 to 24, with higher scores indicating greater utilization of this coping strategy.

^e^ Ranges from 10 to 40, with higher scores indicating greater utilization of this coping strategy.

^f^ Ranges from 12 to 48, with higher scores indicating greater utilization of this coping strategy.

^g^ Ranges from 0 to 84, with higher scores indicating more unmet needs. Only measured at T0 and T1.

### Anxiety and Depression

The intervention group showed a significantly greater reduction in the level of anxiety than the control group at both T1 and T2 with respect to T0 as indicated by the interaction term of group and time (group × T1: B = −3.231 [95% CI, −4.746 to −1.716]; *P* < .001; group × T2: B = −1.890 [95% CI, −3.382 to −0.397]; *P* = .01). Compared with the control group, the intervention group demonstrated a significantly greater reduction in their level of depression at T1 (B = −3.253 [95% CI, −5.052 to −1.454]; *P* < .001) but no significant change at T2 (B = −1.476 [95% CI, −3.259 to 0.308]; *P* = .11).

### QOL

The QOL in both the control and intervention groups declined at T1 and T2 compared with T0, while a significantly slower rate of decline was observed at T1 (B = 13.574 [95% CI, 0.488-26.661]; *P* = .04) but not at T2 (B = 6.236 [95% CI, −7.662 to 20.134]; *P* = .38) in the intervention group compared with the control group. The effects on different dimensions of QOL are displayed in eTable 2 in Supplement 2.

### Coping and Unmet Needs

The intervention group showed an increase in the score for problem-focused and emotional coping and a decrease in dysfunctional coping compared with the control group at T1 and T2 with respect to T0. However, the changes were not statistically significant. The intervention group demonstrated a significantly greater reduction in unmet needs than the control group (B = −12.136 [95% CI, −18.307 to −5.965]; *P* < .001) at T1 relative to T0.

## Discussion

Until now, to our knowledge, no PEI has been developed for the caregivers of AYA patients with cancer. Existing PEIs for caregivers of adult patients with cancer have several shortcomings as they lack consideration of caregivers’ needs and they primarily target caregivers of older adult patients with cancer.^31^ To our knowledge, this study is the first to develop a mobile instant messaging–delivered PEI for caregivers of AYA patients with cancer; thus, it contributes to the understanding of how to support these caregivers and facilitates the evidence-based development of PEIs for caregivers of patients with cancer in general. This study extends previous research and makes a novel contribution to the field.

We found that the mobile instant messaging–delivered PEI met the caregivers’ unmet needs, alleviated their anxiety and depression, and reduced the decline in their QOL immediately after the intervention. A previous study demonstrated similar positive effects on caregivers of adult patients with cancer.^31^ The effects observed in the present study may be explained by the intervention content and the theoretical model adopted. The core content was tailored to the caregivers’ unmet needs, mainly comprising information about AYA cancers and their treatments, illness management at home, communication, self-care, stress management, the regulation of negative emotions, coping with uncertainty, and positive thinking. Thus, it is understandable that the intervention group demonstrated fewer unmet needs, as they engaged in a more positive appraisal process of caregiving.^49^ According to stress and coping theory, a more positive appraisal process leads to better health.^23^ Thus, the intervention group perceived lower levels of anxiety and depression and a better QOL than the control group. However, recognizing that 1 intervention may not address all specific unmet needs, future studies should explore adaptive interventions or provide individualized practical support to address the changing needs of caregivers in clinical practice.^3,50^

The QOL of those in the intervention group declined after the intervention, which is consistent with the findings of 1 previous study^51^ but inconsistent with the findings of other studies.^17,52^ This finding may be because the intervention had minimal focus on caregivers’ physical and spiritual well-being, resulting in a lack of significant effects on these areas. While the intervention improved psychological well-being by facilitating caregiving support, it failed to enhance social well-being due to a lack of specific content addressing this aspect. Consequently, the small effect size was insufficient to counterbalance the overall decline in QOL, which was influenced by disease progression, treatment, and the significantly altered family roles of AYA patients with cancer.^5,53,54^ Future research could consider adding components targeting physical, social, and spiritual well-being to enhance the intervention’s effect on QOL.

Existing evidence for the effects of PEIs on coping is inconclusive.^55,56,57^ The lack of significant changes in coping observed in this study implies that the caregivers may not have acquired effective coping skills. Additional components, such as coping skills training, are needed to help caregivers develop and implement effective coping strategies.^58^ In addition, as shown in Table 3, the intervention group showed improvements in both problem-focused coping and emotion-focused coping from T0 to T1. Thus, we suspect that the lack of significant effects was partly due to the intervention dosage being insufficient to elicit a significant change in coping, as longer interventions and/or more sessions lead to better coping.^16^ Interventions with greater dosages may be needed in future studies to observe a greater effect on coping.

Another important finding was the 12-week sustained effect of the intervention on anxiety but not on other outcomes, which is similar to previous findings related to PEIs for caregivers of adult patients with cancer.^56,59^ The lack of sustained effects on the other outcomes may be mainly due to the continuing and changing support needs of caregivers.^60,61^ This finding indicates that the PEI did not address the caregivers’ changing needs, given that they were no longer able to access the support materials and calls through the instant messaging app once the intervention ended. Therefore, future studies should consider components such as additional counseling, booster sessions, or peer support to enable the caregivers to continue accessing the intervention resources that address their changing needs over a long period.^52,62,63^

### Limitations

This study has several limitations. First, the use of convenience sampling and the recruitment of participants from a single hospital may have limited the generalization of the findings to other regions. Future research should include a multicenter randomized clinical trial to verify the effects and allow greater generalization. Second, all of the outcomes were self-reported without blinding to the treatment group assignment, which potentially introduced biases despite the use of validated instruments and instructing the participants to respond truthfully. Third, the results of the study may only be applicable to caregivers of AYA patients with cancer who are capable of using the mobile instant messaging app. Alternative support methods may be needed for caregivers, such as older adults, who cannot use this app.

## Conclusions

In this randomized clinical trial, the 5-week mobile instant messaging–delivered PEI met the caregivers’ unmet needs, relieved their anxiety and depression, and ameliorated the decline in their QOL immediately after the intervention. Future studies should seek to strengthen the sustained effects of the PEI and explore alternative intervention methods for older adult caregivers of AYA patients with cancer.

## Notes

Supplement 1.
Trial Protocol

Supplement 2. **eAppendix.** Detailed Descriptions of Each Measurement **eTable 1.** Baseline Characteristics Comparison Between Participants Who Completed the Study and Those Who Dropped Out of the Study **eTable 2.** Generalized Estimating Equations Models for Different Dimensions of Quality of Life Across Time Between the Intervention and Control Groups
eReferences

Supplement 3.
Data Sharing Statement
