# Supplementary material for: A Mobile Instant Messaging–Delivered Psychoeducational Intervention for Cancer Caregivers: A Randomized Clinical Trial
Source: JAMA Netw Open. 2024 Feb 22;7(2):e2356522. doi: 10.1001/jamanetworkopen.2023.56522 (PMC10884881; doi:10.1001/jamanetworkopen.2023.56522)
Supplement: Supplement 1. — Trial Protocol [file jamanetwopen-e2356522-s001.pdf]

Supplement 1. Trial protocol

Effects of a Mobile Instant Messaging‑Delivered Psychoeducational Intervention for Cancer Caregivers

Background

Adolescent and young adult (AYA) cancer patients are a segment of the cancer population that warrants additional attention. Various age ranges have been used to define AYA cancer patients, but they are commonly defined as those diagnosed with cancer from 15 to 39 years of age.[1] This definition will be adopted in this study to facilitate comparisons with other studies of AYA cancer patients. In 2020, there were almost 1.2 million AYA cancer cases worldwide, accounting for 6.5% of the total cancer patient population.[2] Of these cases, one fifth (255,316 cases) were reported in China.[2] Although AYA cancer patients constitute a relatively small proportion of the entire cancer population, an increasing trend in AYA cancer incidence rates has been observed in recent decades.[3] In addition, with more than 1 million new cancer diagnoses annually in the AYA population worldwide, the cancer incidence is increasing at a higher rate in this population than in other age groups.[4] These features suggest that AYA cancer patients will constitute an increasing proportion of the total cancer population in the future. Furthermore, the overall 5-year survival rate ranges from 83% to 86% across AYA age groups, which is similar to the survival rate in children (84%), but higher than that in adults over 40 years old (66%).[3] However, the 5-year survival rate varies considerably among cancer types in the AYA population. For example, Hodgkin's lymphoma has a 5-year survival rate of 94%, but acute lymphocytic leukemia has a 5-year survival rate of 60%.[3] Overall, most AYA cancer patients require long-term caregiver support.

Caregivers are the primary source of help and support for patients and are typically family members, partners, or close friends.[5] They also require attention from healthcare providers, as a cancer diagnosis affects both the patients and their caregivers, who can be considered as secondary patients.[6] For caregivers of AYA cancer patients, a cancer diagnosis is a significant source of stress and requires a demanding long-term role.[7,8] Throughout the post-diagnosis course of cancer, the caregivers of AYA cancer patients continue to play crucial roles by offering assistance in daily life activities; communicating with healthcare providers; dealing with medical needs; and providing financial, emotional, and spiritual support to the patients.[7] However, the stress they encounter varies during the different phases of the cancer trajectory, which includes pre-diagnosis, diagnosis, treatment, survivorship, recurrence, and advanced/end-of-life phases.[9] For example, the treatment phase starts from the time of diagnosis and during the initial visit to the outpatient clinic until the last cycle of therapy.[10] The duration of the treatment phase varies based on factors such as the type of cancer, cancer stage, drugs administered, and treatment side effects. Typically, this phase lasts a few months.[10] During this phase, the caregivers confront the unfamiliar scenario of the treatment of the AYA cancer patient. They have to worry about the patients' treatment tolerance and the effectiveness of the treatment.[9] The caregivers also provide physical care to the patients and manage their symptoms,[9] while attempting to strike a balance between their caregiver roles and their other family and social roles.[11] Additionally, the caregivers need to address the AYA patients' unique psychosocial needs, such as fertility preservation, parenting, and education.[1] Therefore, the treatment phase is one of the most stressful phases for caregivers.[9,12]

As they are often under tremendous stress during the treatment of AYA cancer patients, caregivers have many unmet needs. Unmet needs refer to the caregivers' requirements or needs related to problems or issues for which they are unable to obtain assistance.[13] Few caregivers receive training related to their caregiver role,[14] and caregivers consistently report an insufficient level of support available for them, as most resources are directed toward the patients.[15] Consequently, the caregivers of AYA cancer patients often have many unmet needs, mainly psychosocial needs, informational needs, and needs related to practical support and their relationship with the AYA cancer patient.[16] Of the aforementioned needs, these caregivers report the highest demand for informational and emotional support.[17,18] However, evidence is scarce regarding the unmet needs of caregivers of AYA cancer patients in China, where routine support for caregivers is not widely available in most hospitals. It has been reported that only 5.6% of the caregivers of adult cancer patients have no needs or have all their needs met.[19] As cultural differences may effect caregivers' experience in terms of their appraisal of stressful events, perceptions of roles and caregiving responsibilities, perception and use of family support, and coping behaviours,[20,21] it is crucial to explore the unmet needs of caregivers of AYA cancer patients in different cultural contexts.

When caregivers face unmet needs during the caregiving process, they adopt coping strategies to deal with their situation.[22] The specific coping strategies used can vary depending on the context.[23] One study found that the caregivers of AYA cancer patients were more likely to adopt coping strategies,

such as problem solving, cognitive restructuring, expressing emotions, and social contract, than other strategies, including problem avoidance, wishful thinking, self-criticism, and social withdrawal.[24] Nevertheless, most caregivers demonstrate inadequate coping strategies.[25]

According to stress and coping theory, an individual's health is affected by their appraisal, coping, and interactive processes during stressful events.[23] Of the various factors that affect caregivers' appraisal of stress, unmet needs are particularly important.[26] Consequently, caregivers' unmet needs and inadequate coping negatively influence their health.[27,28] Previous studies have found that approximately one in three caregivers of AYA cancer patients have moderately to severely elevated anxiety and depressive symptoms.[29,30] Caregivers have also reported reduced quality of life after taking care of patients.[31]

Given the needs of caregivers of AYA cancer patients and the negative effects that they experience, support for these caregivers is essential. The National Cancer Institute recently emphasized the importance of developing interventions tailored to the caregivers of AYA patients, given their unique life course and social needs.[32] However, the majority of currently available interventions focus on the caregivers of older adult cancer patients, because the prevalence of cancer is higher in the population aged above 40 years.[32] Few interventions are specifically designed for the caregivers of AYA cancer patients. Therefore, additional research on interventions for caregivers of AYA cancer patients is warranted to address their unmet needs and improve their health.

Psychoeducational interventions (PEIs) are commonly used interventions to address the needs of and challenges faced by caregivers of cancer patients.[33] Given the various unmet needs reported by caregivers of AYA cancer patients, particularly their informational and emotional needs,[17,18] a PEI may be a suitable type of intervention, as it provides information and deals with the emotional and psychosocial needs of caregivers.[33] Previous studies have shown that a PEI is a cost-effective strategy to improve the outcomes of caregivers of cancer patients, such as meeting their needs, relieving their psychological stress, and enhancing their quality of life.[34-38] However, to the best of our knowledge, there has been no research on PEIs for AYA cancer patients. The content of PEIs for caregivers of cancer patients in other age groups may not be directly applicable to caregivers of AYA cancer patients.[39] In addition, caregivers' suboptimal health needs to be addressed. Therefore, in this study, we will develop a mobile instant messaging-delivered PEI for caregivers of AYA cancer patients and examine its effectiveness.

Methods

Design

A two-arm (parallel-group) randomized controlled trial with repeated measurements will be conducted. Eligible caregivers of AYA cancer patients will be invited to participate. After their written informed consent is obtained and baseline data collection is completed, the participants will be randomly allocated to the intervention or control group at a 1:1 ratio using a randomized block design, with block sizes of four or six. Sequentially numbered, opaque, sealed envelopes will be used to guarantee allocation concealment. The participants in the intervention group will receive the mobile instant messaging-delivered PEI and usual care, while those in the control group will receive usual care. The outcomes (anxiety, depression, quality of life and coping) will be reassessed 5 weeks (immediately after the intervention) and 12 weeks after baseline. Unmet needs will be reassessed 5 weeks after baseline. A repeated measurement design of three time points with 12 weeks of follow-up will be used, because a 12-week follow-up period has been shown to be sufficient to observe changes in the outcomes of interest in this study.[40] The outcomes will be remeasured 5 weeks after baseline (immediately after the intervention) to evaluate whether the intervention had any immediate effects.

Setting and Participants

Setting

The study participants will be recruited from the inpatient wards of a tertiary cancer hospital in Changsha, Hunan Province, China.

Sampling Method

Potential participants will be the caregivers of AYA cancer patients in the treatment phase. They will be recruited using convenience sampling and included if they: 1) caregivers caring for AYA cancer patients during the treatment phase; 2) are still caring for the patients after discharge; 3) aged at least 18

years; 4) caregivers who have at least one item with a high level of unmet need in the needs assessment; 5) can understand the research project and can read and communicate in Chinese; 6) are reachable via the mobile instant messaging application (WeChat); and 7) provide informed consent to participate in this study. If two or more caregivers are available for a given patient, the one undertaking the primary caregiving responsibility will be included. The principal investigator (PI) or the research assistants, who are responsible for recruiting, will ask the doctors about the expected duration of treatment for the AYA cancer patients, to ensure that they will be undergoing treatment throughout the study period. Caregivers excluded if: 1) they are being paid; 2) the AYA cancer patient under their care is receiving hospice care; 3) they are mentally unstable or have cognitive impairments that result in an inability to participate in the intervention; or 4) they are participating in another research interventional program.

Sample Size Planning

The sample size was calculated based on the primary outcomes (anxiety and depression) using G-power (version 3.1.9.7; Heinrich-Heine-Universität Düsseldorf, Düsseldorf, Germany).[41] Considering that no similar study has been conducted for caregivers of AYA cancer patients, the effect sizes of the primary outcomes in previous studies of PEIs for caregivers of adult cancer patients were used to calculate the sample size. The combined effect size for anxiety in previous studies was −0.50.[40] For a two-tailed α error of 5%, 80% power, and a test of two independent groups, the required sample size for each group was calculated as 64. An acceptable attrition rate has been shown to be less than 20%.[42,43] Thus, we assumed an attrition rate of 20% for this study. As a result, the final sample size was calculated as 160 for the two arms. The effect size for depression in previous studies was −0.54.[40] Based on this effect size, the final sample size was calculated as 138, when using the same parameters as those used for the anxiety outcome. We adopted a larger sample size to guarantee sufficient statistical power. Therefore, the required sample size in this study is 160, with 80 in each group.

Intervention

Control Group

The participants in the control group will receive usual care, including routine inpatient and discharge education. The hospital's telephone helpline number will also be provided.

Intervention Group

Participants in the intervention group will receive the 5-week mobile instant messaging-delivered PEI, which was developed by the PI and the research team based on an assessment of caregivers' unmet needs in our previous study.[44] Stress and coping theory was used as the theoretical framework for the development of this intervention.[45] According to this theory, the intervention content will be guided by two coping strategies (problem-based coping and emotion-based coping) based on the caregivers' unmet needs, including worries about the future, informational needs, personal and emotional needs, needs for healthcare access and continuity, and financial needs.[44] To enhance the problem-focused coping strategies, the intervention will provide information to address the caregivers' informational needs, financial needs, and needs for healthcare access and continuity. Emotion-focused strategies function by regulating emotions, modifying the way stress interacts with the environment, or changing the relational meaning of what is happening.[46] Thus, the intervention will mainly educate the caregivers about strategies to cope with their worries about the future and their personal and emotional needs.

Caregivers' informational needs are mainly related to the patient's disease, common treatments, treatment side effects, managing the illness at home, communicating with healthcare professionals, and talking about the illness with the patient. The financial needs mainly involve their need to pay non-medical costs and to receive financial assistance. As we cannot provide direct financial assistance to the caregivers, information about medical insurance and relevant financial assistance programs will be provided. These need-oriented contents will be delivered over five weekly sessions, based on what the caregivers may need each week. For example, in the first week, when the caregivers may need to care for the patients in the hospital, the intervention contents will mainly aim to help the caregivers deal with the medical needs of the patient during their stay in the hospital. Information about the patient's disease, treatments, and treatment side effects; medical insurance and financial assistance programs; how to obtain medical services; and how to communicate with healthcare professionals effectively will be provided in the first week. The contents of each session of the PEI with respect to unmet needs are presented in Table 1.

The whole intervention will be delivered by the PI to ensure intervention consistency. The five sessions of the intervention will be conducted via a mobile instant messaging application, the most popular social media platform in China. This platform allows free instant messages to be sent to individuals or groups through articles and video calls. The first session will be initiated within 1 week

of caregivers recruitment. The following four sessions will be delivered at weekly intervals. At the beginning of each session, a short briefing about the purpose and process of the session will be provided. Towards the end of each session, the intervenor will answer the caregivers' questions and ensure that the caregivers do not have any problems with the content of the current session. At the end of each session, the intervenor will make an appointment with the caregivers for the next session. For sessions one to four, the educational content will be distributed in the form of articles through instant messaging, which are deemed as an effective, sustainable, and feasible strategy for health education.[47] During each session, the PI will send the session-specific articles to the caregivers through instant messaging. The PI will then contact individual participants through video calls via the mobile instant messaging application. During the calls, the PI will use the teach-back strategy to confirm that the participants have read and understood the information provided in the article and will clarify any misunderstandings. The PI will first ask the participants to explain what they have learned in their own words. If there are any misunderstandings, the PI will clarify them. The PI will also discuss the caregivers' problems or concerns with respect to the contents of each session and discuss possible strategies to overcome these problems. The caregivers will be encouraged to express their emotions and concerns throughout the process. In the fifth session, a follow-up call via the mobile instant messaging application will be made to each caregiver to determine whether they have any other unmet needs and if so, they will be able to discuss them.

Table 1 The mobile instant messaging-delivered psychoeducational intervention for caregivers of adolescent and young adult cancer patients

Session	Coping functions	Needs	Content	Duration
**Session 1**	Problem-based coping	Information, financial, and healthcare access and continuity	• Provide information on cancer, treatments and side effects<br>• Provide information on medical insurance and financial assistance programmes<br>• Teach the caregivers how to obtain healthcare services<br>• Teach the caregivers how to communicate with the healthcare professionals effectively	30–45 min
**Session 2**		Information	• Provide information on AYA populations and their fertility issues<br>• Educate the caregivers on how to help manage the patients' illness at home<br>• Educate the caregivers on how to communicate and talk about the illness with the patients	30–45 min
**Session 3**	Emotion-based coping	Personal and emotional	• Teach the caregivers how to care for themselves<br>• Encourage the caregivers to share their stress and emotions<br>• Teach the caregivers how to manage their stress<br>• Teach the caregivers how to regulate negative emotions	30–45 min
**Session 4**		Worries about the future	• Discuss the caregivers' uncertainties or worries<br>• Teach the caregivers how to cope with uncertainty<br>• Encourage optimistic thinking	30–45 min
**Session 5**		Other needs	• Discuss the caregivers' other needs	10–20 min

Outcome Measurements

Sociodemographic and Clinical Characteristics

We will use a structured data collection form to collect the caregivers' sociodemographic characteristics (i.e. sex, age, educational level, marital status, place of residence, employment, family income, and relationship with the patient) and the patients' sociodemographic characteristics (i.e. sex, age, and type of medical insurance) and clinical characteristics (i.e. type of cancer, age when diagnosed, stage of cancer, and current cancer treatment).

Primary Outcomes

Anxiety

Anxiety will be measured using the 7-item Generalised Anxiety Disorder Scale (GAD-7), which is a self-reported scale consisting of seven items that measure the respondents' experience of anxiety over the previous 2 weeks. The response options range from "not at all (0)" to "nearly every day (3)". The total score ranges from 0 to 21, with higher scores indicating higher levels of anxiety.[48,49] The Chinese version of the GAD-7 has demonstrated good internal consistency (Cronbach's alpha = 0.898) and test–retest reliability (intraclass correlation coefficient [ICC] = 0.856).[50] It also has good convergent validity with the Hospital Anxiety and Depression Scale and the Hamilton Depression Scale.[50] The GAD-7 has been widely used to assess caregivers of cancer patients.[51-53]

Depression

Depression will be measured using the Patient Health Questionnaire-9 (PHQ-9). This is an instrument used to measure the severity of depression in the general population during the past 2 weeks. A 4-point scale is used to rate the degree of severity from "not at all (0)" to "nearly every day (3)". The total score of the PHQ-9 ranges from 0 to 27, with higher scores indicating higher levels of depression.[54,55] The simplified Chinese version has shown good internal consistency (Cronbach's alpha = 0.857) and test–retest reliability (ICC= 0.857).[56] It also has good convergent validity with the Hospital Anxiety and Depression Scale and the Hamilton Depression Scale.[56] The scale is widely used to assess the level of depression in caregivers of cancer patients.[51-53]

Secondary Outcomes

Quality of life

Caregivers' quality of life will be assessed using the Quality of Life Scale—Family Version (QOL-Scale FAM).[57] The Chinese version has 35 items and four dimensions. Respondents rate each item on a linear scale ranging from 0 to 10. The total score ranges from 0 to 350, with higher scores implying better quality of life.[58] The Chinese version of the QOL-Scale FAM has good reliability and validity, with a Cronbach's α value of 0.794 for the total questionnaire. It has been used widely to assess the caregivers of patients with various types of cancer.[59-61]

Coping

The caregivers' level of coping will be evaluated using the Brief Coping Orientations to Problems Experienced Scale (Brief-COPE).[62,63] It comprises 28 items that can be categorized into three types of coping strategies: problem-focused strategies, emotion-focused strategies, and dysfunctional coping strategies.[62] Each item is rated on a 4-point Likert scale ranging from "I haven't been doing this at all (1)" to "I've been doing this a lot (4)". No overall score is available for the total scale. The instructions for the tool advise adding the scores for each subscale separately to determine the respondents' usage of each particular coping strategy. A higher score represents greater utilization of a specific coping strategy. The Chinese version of Brief-COPE has sound reliability,[63] and has been widely used to evaluate the coping methods of caregivers of cancer patients.[64,65]

Unmet needs

The Support Person's Unmet Needs Survey—Short Form (SPUNS-SF) will be used to measure the caregivers' unmet needs.[66,67] The Chinese version is a 21-item validated scale used to measure caregivers' unmet needs, including informational needs, worries about the future, financial needs, the need for healthcare access and continuity, and personal and emotional needs.[67] All items are rated with a score of 0 to 4, and a score of 3 or above is classified as a "high-level" unmet need.[66] The scores for all items are summed to calculate the total score, with a higher score representing a higher level of unmet need. The SPUNS-SF has been validated in 1,026 Chinese caregivers of cancer patients and showed good reliability and validity.[67] The internal consistency of the Chinese version of the

SPUNS-SF is high, with a Cronbach's alpha coefficient of 0.94.[67]

Data Collection Procedures

Before baseline data collection, the PI and the research assistants responsible for participant recruitment will assess the unmet needs of the potential participants to determine their eligibility. The eligible participants who consent to participate in the study will be invited to complete questionnaires to perform the baseline assessment. The participants will complete their baseline assessment by filling out paper questionnaires on-site. Follow-up assessments will then be conducted 5 weeks (T1) and 12 weeks (T2) after the baseline assessment. e-Questionnaires will be used for follow-up data collection. The contents of the corresponding questionnaires at each follow-up time point will be input into an e-questionnaire platform (https://www.wjx.cn/). The link containing these questionnaires will then be sent to each participant individually through the mobile instant messaging application. The participants will complete the questionnaires independently, given that all questionnaires used in this study are self-reported. To facilitate the participants returning the e-questionnaires on time, the PI will send reminder messages if the questionnaire is not completed on that day. In addition, they will be asked to enter the study ID assigned to them, instead of their names, to protect their privacy. The PI will determine who has completed the questionnaire by identifying their unique study ID. After the participants complete the questionnaires, the PI will thoroughly review the responses for any instances of duplicate records or consecutively consistent answers. If the participants provide consistent answers for ten or more consecutive questions, it will be deemed as straightlining.[68] To ensure the accuracy of the responses in such cases, a research assistant, who is unaware of the intervention allocation, will contact the participants and confirm their responses to these questions.

Data Analysis

All data will be analyzed using SPSS 26.0 (IBM Corp., Armonk, NY, USA). Continuous data will be tested for normality by assessing skewness and kurtosis and by a visual inspection of Q-Q plots.[69,70] The sociodemographic data and the caregivers' outcomes will be presented using descriptive statistics, as appropriate. The data will be described as means and standard deviations if normally distributed or medians and interquartile ranges if non-normally distributed. Categorical variables will be expressed as numbers (n) and percentages (%). The skewed variables will be appropriately transformed as necessary before testing the intervention effects. A generalized estimating equations (GEE) model will be used to explore the effects of the mobile instant messaging-delivered PEI on the outcomes of interest in this study. The effects of the intervention on the outcome variables will be compared across three time points, namely, T0 (baseline), T1 (5 weeks after baseline), and T2 (12 weeks after baseline) or two-time points (T0 and T1), as appropriate. The control group (assigned as 0) and the baseline (assigned as 0) will be set as the references in the GEE model. The intention-to-treat principle will be followed.[71] The homogeneity between the participants who complete the study and those who drop out will be compared using an independent Student's t-test, Mann–Whitney U test, chi-square test, or Fisher's exact test, as appropriate. If the missing data are deemed to be missing at random, they will be left unaddressed, because GEE models allow data to be missing at random and yield valid results by analyzing the observed data.[72] Otherwise, multiple imputation will be used to impute the missing data.

Ethical Considerations

Ethics approval will be obtained from the Joint Chinese University of Hong Kong–New Territories East Cluster Clinical Research Ethics Committee (The Joint CUHK-NTEC CREC) and the hospital where the study will be conducted.

Each stage of the study will follow the ethical principles of the Declaration of Helsinki.[73] All of the potential participants of this study will be informed of the purpose, content, and potential benefits and harms of the research. The potential participants will have the right to ask questions about the study and be fully informed. Participation in this study will be totally voluntary and the decision whether to participate will not affect the treatment of the patients. The participants will have the right to withdraw from the study at any time. Written consent will be obtained from each participant and confidentiality will be assured. All information pertaining to individuals participating in this study will be anonymized from the outset of the study. Code numbers will be used for data collection and statistical analysis. All information collected in this study will be kept confidential and used only for research purposes and the research data will be stored in a secure manner. The paper questionnaires will be kept in a locked cabinet and the e-questionnaires will only be accessible to the researcher. The data in SPSS will be stored in a file protected by a password that is only known by the researcher. Therefore, all research data will only be accessible to the researcher. We will not publish any of the participants' personal information when we analyze our data and publish our research results. After the

completion of the study, the data will be saved for 5 years. After that, it will be erased from the computer and the paper documents will be destroyed.

Impact of the Study on Nursing Practice

To the best of our knowledge, this will be the first study to develop and apply a PEI for caregivers of AYA cancer patients. Because of the growing number of caregivers supporting AYA cancer patients and the special needs of these patients, an intervention tailored to the unique needs and situations of these caregivers is needed. However, there is little research on interventions specific to the caregivers of AYA cancer patients. As a common intervention for caregivers of cancer patients, a PEI for caregivers of AYA cancer patients has the potential to meet the caregivers' needs and enhance their coping, further improving their health. This study will contribute to the knowledge of how to support the caregivers of AYA cancer patients and will facilitate the evidence-based development of interventions for caregivers of cancer patients in general.

In addition, the nurse-led PEI evaluated in this study will provide scientific evidence for the delivery of PEIs in clinical practice by nurses. Nurses are the most common intervenors who deliver PEIs to caregivers. They are also the healthcare providers who have the closest contact with the caregivers of AYA cancer patients, and as such, they can build rapport with the caregivers, which facilitates delivery of the PEI. Therefore, nurses are in an advantageous position to deliver PEIs for caregivers. The experience of implementing the nurse-led PEI in this study will provide a reference for clinical practice. To conclude, the study has significance for both knowledge development in nursing research and clinical practice.

References

1. National Comprehensive Cancer Network. *Adolescent and Young Adult (AYA) Oncology.* 2021.
2. International Agency for Research on Cancer. Estimated number of new cases in 2020, all cancers, both sexes, ages 15-39. https://gco.iarc.fr/today/online-analysis-table?v=2020&mode=population&mode_population=countries&population=900&populations=900&key=asr&sex=0&cancer=39&type=0&statistic=5&prevalence=0&population_group=0&ages_group%5B%5D=3&ages_group%5B%5D=7&group_cancer=1&include_nmsc=1&include_nmsc_other=1. Published 2020. Accessed May 6, 2021, 2021.
3. Miller KD, Fidler-Benaoudia M, Keegan TH, Hipp HS, Jemal A, Siegel RL. Cancer statistics for adolescents and young adults, 2020. *Ca-A Cancer Journal for Clinicians.* 2020;70(6):443–459.doi: 10.3322/caac.21637
4. Bleyer A, Ferrari A, Whelan J, Barr RD. Global assessment of cancer incidence and survival in adolescents and young adults. *Pediatric Blood & Cancer.* 2017;64(9):e26497.doi: 10.1002/pbc.26497
5. Alfano CM, Leach CR, Smith TG, et al. Equitably improving outcomes for cancer survivors and supporting caregivers: A blueprint for care delivery, research, education, and policy. *Ca-A Cancer Journal for Clinicians.* 2019;69(1):35–49.doi: 10.3322/caac.21548
6. Williams AL. Family Caregivers to Adults with Cancer: The Consequences of Caring. *Recent Results in Cancer Research.* 2018;210:87–103.doi: 10.1007/978-3-319-64310-6_6
7. Ferrell BR, Kravitz K. Cancer care: Supporting underserved and financially burdened family caregivers. *Journal of the Advanced Practitioner in Oncology.* 2017;8(5):494–500.doi: 10.6004/jadpro.2017.8.5.5
8. Juth V, Silver RC, Sender L. The shared experience of adolescent and young adult cancer patients and their caregivers. *Psycho-oncology.* 2015;24(12):1746–1753.doi: 10.1002/pon.3785
9. Northouse LL, Katapodi MC, Schafenacker AM, Weiss D. The impact of caregiving on the psychological well-being of family caregivers and cancer patients. *Seminars in Oncology Nursing.* 2012;28(4):236–245.doi: 10.1016/j.soncn.2012.09.006
10. Grov EK. The cancer trajectory—a model of phases. *Vård i Norden.* 2014;34(1):46–47.doi.
11. Schulz R, Eden J, National Academies of Sciences E, Medicine. Family caregiving roles and impacts. In: Schulz R, Eden J, eds. *Families caring for an aging America.* National Academies Press (US); 2016.
12. Nijboer C, Tempelaar R, Sanderman R, Triemstra M, Spruijt RJ, van den Bos GA. Cancer and caregiving: the impact on the caregiver's health. *Psycho-oncology.* 1998;7(1):3–13.doi: 10.1002/(SICI)1099-1611(199801/02)7:1<3::AID-PON320>3.0.CO;2-5
13. Campbell HS, Sanson-Fisher R, Taylor-Brown J, Hayward L, Wang XS, Turner D. The cancer support person's unmet needs survey: psychometric properties. *Cancer.* 2009;115(14):3351–3359.doi: 10.1002/cncr.24386
14. Reblin M, Ketcher D, Vadaparampil ST. Care for the cancer caregiver: A qualitative study of facilitators and barriers to caregiver integration and support. *Journal of Cancer Education.* 2021;37(6):1634–1640.doi: 10.1007/s13187-021-02001-6
15. Nicklin E, Velikova G, Hulme C, et al. Long-term issues and supportive care needs of adolescent and young adult childhood brain tumour survivors and their caregivers: A

systematic review. *Psycho-oncology.* 2019;28(3):477–487.doi: 10.1002/pon.4989

16. Carey ML, Clinton-McHarg T, Sanson-Fisher RW, Shakeshaft A. Development of cancer needs questionnaire for parents and carers of adolescents and young adults with cancer. *Supportive Care in Cancer.* 2012;20(5):991–1010.doi: 10.1007/s00520-011-1172-2
17. McCarthy MC, McNeil R, Drew S, Orme L, Sawyer SM. Information needs of adolescent and young adult cancer patients and their parent-carers. *Supportive Care in Cancer.* 2018;26(5):1655–1664.doi: 10.1007/s00520-017-3984-1
18. Sawyer SM, McNeil R, McCarthy M, et al. Unmet need for healthcare services in adolescents and young adults with cancer and their parent carers. *Supportive Care in Cancer.* 2017;25(7):2229–2239.doi: 10.1007/s00520-017-3630-y
19. Niu A, Guo C, Zhong D, et al. Identifying the unmet supportive care needs, with concomitant influencing factors in family caregivers of cancer patients in China. *Asia-Pacific Journal of Oncology Nursing.* 2021;8(3):276–286.doi: 10.4103/apjon.apjon_52_20
20. Aranda MP, Knight BG. The influence of ethnicity and culture on the caregiver stress and coping process: a sociocultural review and analysis. *Gerontologist.* 1997;37(3):342–354.doi: 10.1093/geront/37.3.342
21. Dilworth-Anderson P, Williams IC, Gibson BE. Issues of race, ethnicity, and culture in caregiving research: a 20-year review (1980-2000). *Gerontologist.* 2002;42(2):237–272.doi: 10.1093/geront/42.2.237
22. Girgis A, Lambert SD, McElduff P, et al. Some things change, some things stay the same: a longitudinal analysis of cancer caregivers' unmet supportive care needs. *Psycho-oncology.* 2013;22(7):1557–1564.doi: 10.1002/pon.3166
23. Lazarus RS, Folkman S. *Stress, appraisal, and coping.* New York: Springer Publishing Company; 1984.
24. Hodgson J, Lamson A, Kolobova I, et al. The experience of distress and coping among young adults with cancer and their caregivers. *Contemporary Family Therapy.* 2022;44(3):199–209.doi: 10.1007/s10591-021-09592-8
25. Given B, Sherwood PR. Family care for the older person with cancer. *Seminars in Oncology Nursing.* 2006;22(1):43–50.doi: 10.1016/j.soncn.2005.10.006
26. Wang S, Cheung DSK, Leung AYM, Davidson PM. Factors associated with caregiving appraisal of informal caregivers: A systematic review. *Journal of Clinical Nursing.* 2020;29(17-18):3201–3221.doi: 10.1111/jocn.15394
27. Friethriksdottir N, Saevarsdottir T, Halfdanardottir SI, et al. Family members of cancer patients: Needs, quality of life and symptoms of anxiety and depression. *Acta Oncologica.* 2011;50(2):252–258.doi: 10.3109/0284186X.2010.529821
28. Buzgova R, Spatenkova N, Fukasova-Hajnova E, Feltl D. Assessing needs of family members of inpatients with advanced cancer. *European Journal of Cancer Care.* 2016;25(4):592–599.doi: 10.1111/ecc.12441
29. Mikrut EE, Panjwani AA, Cipollina R, Revenson TA. Emotional adjustment among parents of adolescents and young adults with cancer: The influence of social constraints on cognitive processing and fear of recurrence. *Journal of Behavioral Medicine.* 2020;43(2):237–245.doi: 10.1007/s10865-019-00072-x
30. McCarthy MC, McNeil R, Drew S, et al. Psychological distress and posttraumatic stress symptoms in adolescents and young adults with cancer and their Parents. *Journal of*

Adolescent and Young Adult Oncology. 2016;5(4):322–329.doi: 10.1089/jayao.2016.0015

31. Panjwani AA, Millar BM, Revenson TA. Tolerating uncertainty in the dark: Insomnia symptoms, distress, and well-being among parents of adolescents and young adults with cancer. *International Journal of Behavioral Medicine.* 2021;28(1):14–20.doi: 10.1007/s12529-020-09869-6
32. Kent EE, Mollica MA, Buckenmaier S, Wilder Smith A. The characteristics of informal cancer caregivers in the United States. *Seminars in Oncology Nursing.* 2019;35(4):328–332.doi: 10.1016/j.soncn.2019.06.002
33. Northouse LL, Katapodi MC, Song L, Zhang L, Mood DW. Interventions with family caregivers of cancer patients: meta-analysis of randomized trials. *Ca-A Cancer Journal for Clinicians.* 2010;60(5):317–339.doi: 10.3322/caac.20081
34. Leow M, Chan S, Chan M. A pilot randomized, controlled trial of the effectiveness of a psychoeducational intervention on family caregivers of patients with advanced cancer. *Oncology nursing forum.* 2015;42(2):E63-72.doi: 10.1188/15.Onf.E63-e72
35. Hudson P, Trauer T, Kelly B, et al. Reducing the psychological distress of family caregivers of home based palliative care patients: longer term effects from a randomised controlled trial. *Psycho-oncology.* 2015;24(1):19-24.doi: 10.1002/pon.3610
36. Holm M, Årestedt K, Carlander I, et al. Short-term and long-term effects of a psycho-educational group intervention for family caregivers in palliative home care - results from a randomized control trial. *Psycho-oncology.* 2016;25(7):795–802.doi: 10.1002/pon.4004
37. Hudson PL, Aranda S, Hayman-White K. A psycho-educational intervention for family caregivers of patients receiving palliative care: a randomized controlled trial. *Journal of pain and symptom management.* 2005;30(4):329-341.doi: 10.1016/j.jpainsymman.2005.04.006
38. Hudson PL, Trauer T, Lobb E, et al. Supporting family caregivers of hospitalised palliative care patients: a psychoeducational group intervention. *BMJ Supportive & Palliative Care.* 2012;2(2):115–120.doi: 10.1136/bmjspcare-2011-000131
39. Bonell C, Oakley A, Hargreaves J, Strange V, Rees R. Assessment of generalisability in trials of health interventions: suggested framework and systematic review. *BMJ.* 2006;333(7563):346–349.doi: 10.1136/bmj.333.7563.346
40. Cheng Q, Xu B, Ng MSN, Duan Y, So WKW. Effectiveness of psychoeducational interventions among caregivers of patients with cancer: A systematic review and meta-analysis. *International Journal of Nursing Studies.* 2022;127:104162.doi: 10.1016/j.ijnurstu.2021.104162
41. Faul F, Erdfelder E, Buchner A, Lang AG. Statistical power analyses using G*Power 3.1: tests for correlation and regression analyses. *Behavior Research Methods.* 2009;41(4):1149–1160.doi: 10.3758/BRM.41.4.1149
42. Gourash WF, Lockhart JS, Kalarchian MA, Courcoulas AP, Nolfi D. Retention and attrition in bariatric surgery research: an integrative review of the literature. *Surgery for Obesity and Related Diseases.* 2016;12(1):199–209.doi: 10.1016/j.soard.2015.09.006
43. Gul RB, Ali PA. Clinical trials: the challenge of recruitment and retention of participants. *Journal of Clinical Nursing.* 2010;19(1-2):227–233.doi: 10.1111/j.1365-2702.2009.03041.x
44. Cheng Q, Ng MSN, Choi KC, So WKW. Unmet needs, anxiety, depression, and quality of life among caregivers of adolescents and young adults with cancer: A cross-sectional study.

Asia-Pacific Journal of Oncology Nursing. 2022;9(10):100108.doi: 10.1016/j.apjon.2022.100108

45. Lazarus RS. *Stress, appraisal, and coping [electronic resource].* New York: Springer Pub. Co.; 1984.
46. Lazarus RS. Coping theory and research: past, present, and future. *Psychosomatic Medicine.* 1993;55(3):234–247.doi: 10.1097/00006842-199305000-00002
47. Sun M, Yang L, Chen W, et al. Current status of official WeChat accounts for public health education. *Journal of Public Health.* 2021;43(3):618–624.doi: 10.1093/pubmed/fdz163
48. Lowe B, Decker O, Muller S, et al. Validation and standardization of the Generalized Anxiety Disorder Screener (GAD-7) in the general population. *Medical Care.* 2008;46(3):266–274.doi: 10.1097/MLR.0b013e318160d093
49. Spitzer RL, Kroenke K, Williams JB, Lowe B. A brief measure for assessing generalized anxiety disorder: the GAD-7. *Archives of Internal Medicine.* 2006;166(10):1092–1097.doi: 10.1001/archinte.166.10.1092
50. He X, Li C, Qian J, Cui H, Wu W. Reliability and validity of a generalized anxiety disorder scale in general hospital outpatients. *Shanghai Archives of Psychiatry.* 2010;22(4):200–203.doi.
51. Parker Oliver D, Washington K, Smith J, Uraizee A, Demiris G. The prevalence and risks for depression and anxiety in hospice caregivers. *Journal of Palliative Medicine.* 2017;20(4):366–371.doi: 10.1089/jpm.2016.0372
52. Ullrich A, Ascherfeld L, Marx G, Bokemeyer C, Bergelt C, Oechsle K. Quality of life, psychological burden, needs, and satisfaction during specialized inpatient palliative care in family caregivers of advanced cancer patients. *BMC Palliative Care.* 2017;16(1):31.doi: 10.1186/s12904-017-0206-z
53. Oechsle K, Ullrich A, Marx G, et al. Psychological burden in family caregivers of patients with advanced cancer at initiation of specialist inpatient palliative care. *BMC Palliative Care.* 2019;18(1):102.doi: 10.1186/s12904-019-0469-7
54. Martin A, Rief W, Klaiberg A, Braehler E. Validity of the Brief Patient Health Questionnaire Mood Scale (PHQ-9) in the general population. *General Hospital Psychiatry.* 2006;28(1):71–77.doi: 10.1016/j.genhosppsych.2005.07.003
55. Kroenke K, Spitzer RL, Williams JB. The PHQ-9: Validity of a brief depression severity measure. *Journal of General Internal Medicine.* 2001;16(9):606–613.doi: 10.1046/j.1525-1497.2001.016009606.x
56. Bian C, He X, Qian J, Wu W, Li C. The reliability and validity of a modified patient health questionnaire for screening depressive syndrome in general hospital outpatients. *Journal of Tongji University (Medical Science).* 2009;30(5):136-140.doi.
57. Ferrell BR, Grant M. Quality of Life Family Version. https://www.cityofhope.org/doc/1431763601545-qol-family.pdf. Published 2005. Accessed.
58. Liu Y, Gan X, Tang W, Li X. Application of QOL Scale-FAMILY in Evaluation of Quality of Life for Caregivers of Patients with Laryngocarcinoma. *Chinese General Practice.* 2009;12(5A):726-728.doi: 1007-9572(2009) 05-0726-03
59. Jin M, Li L, Yao J. Role of social support in the quality of life and burden of care of primary caregivers of patients with liver cancer. *Nursing Journal of Chinese People's Liberation Army.* 2020;37(05):65-68+75.doi.

60. Luan B, Wang W, Zhu Y. The relationship between the care burden and quality of life among caregivers of elderly cancer patients receiving chemotherapy. *Chinese Journal of Gerontology.* 2017;37(20):5162-5164.doi.
61. Wu L, Gao J, Gu J, Yu Y, Jiang L. Effect of family intervention on caregiver burden and quality of life of lung cancer patients. *Shanghai Nursing.* 2020;20(09).doi.
62. Carver CS. You want to measure coping but your protocol's too long: Consider the brief COPE. *International Journal of Behavioral Medicine.* 1997;4(1):92–100.doi: 10.1207/s15327558ijbm0401_6
63. Wang XQ, Lambert CE, Lambert VA. Anxiety, depression and coping strategies in post-hysterectomy Chinese women prior to discharge. *International Nursing Review.* 2007;54(3):271–279.doi: 10.1111/j.1466-7657.2007.00562.x
64. Han Y, Hu D, Liu Y, et al. Coping styles and social support among depressed Chinese family caregivers of patients with esophageal cancer. *European Journal of Oncology Nursing.* 2014;18(6):571–577.doi: 10.1016/j.ejon.2014.07.002
65. Wang T, Molassiotis A, Tan JY, Chung BPM, Huang HQ. Prevalence and correlates of unmet palliative care needs in dyads of Chinese patients with advanced cancer and their informal caregivers: a cross-sectional survey. *Supportive Care in Cancer.* 2021;29(3):1683–1698.doi: 10.1007/s00520-020-05657-w
66. Campbell SH, Carey M, Sanson-Fisher R, et al. Measuring the unmet supportive care needs of cancer support persons: the development of the support person's unmet needs survey--short form. *European Journal of Cancer Care.* 2014;23(2):255–262.doi: 10.1111/ecc.12138
67. Han Y, Zhou Y, Wang J, et al. Chinese version of the Cancer Support Person's Unmet Needs Survey-Short Form: A psychometric study. *European Journal of Cancer Care.* 2019;28(2):e12963.doi: 10.1111/ecc.12963
68. Schonlau M, Toepoel V. Straightlining in Web survey panels over time. *Survey Research Methods.* 2015;9(2):125–137.doi: 10.18148/srm/2015.v9i2.6128
69. Kim HY. Statistical notes for clinical researchers: assessing normal distribution (2) using skewness and kurtosis. *Restorative Dentistry & Endodontics.* 2013;38(1):52–54.doi: 10.5395/rde.2013.38.1.52
70. Mishra P, Pandey CM, Singh U, Gupta A, Sahu C, Keshri A. Descriptive statistics and normality tests for statistical data. *Annals of Cardiac Anaesthesia.* 2019;22(1):67–72.doi: 10.4103/aca.ACA_157_18
71. Montori VM, Guyatt GH. Intention-to-treat principle. *Canadian Medical Association Journal.* 2001;165(10):1339–1341.doi.
72. Seaman S, Copas A. Doubly robust generalized estimating equations for longitudinal data. *Statistics in Medicine.* 2009;28(6):937–955.doi: 10.1002/sim.3520
73. World Medical Association. WMA Declaration of Helsinki - Ethical Principles for Medical Research Involving Human Subjects. https://www.wma.net/policies-post/wma-declaration-of-helsinki-ethical-principles-for-medical-research-involving-human-subjects/. Published 2018. Accessed 2021.8.27, 2021.
