# Supplementary material for: A Mobile Instant Messaging–Delivered Psychoeducational Intervention for Cancer Caregivers: A Randomized Clinical Trial
Source: JAMA Netw Open. 2024 Feb 22;7(2):e2356522. doi: 10.1001/jamanetworkopen.2023.56522 (PMC10884881; doi:10.1001/jamanetworkopen.2023.56522)
Supplement: Supplement 2. — eAppendix. Detailed Descriptions of Each Measurement eTable 1. Baseline Characteristics Comparison Between Participants Who Completed the Study and Those Who Dropped Out of the Study eTable 2. Generalized Estimating Equations Models for Different Dimensions of Quality of Life Across Time Between the Intervention and Control Groups eReferences [file jamanetwopen-e2356522-s002.pdf]

Supplementary Online Content

Cheng Q, Ng MSN, Choi KC, Chen Y, Liu G, So WKW. Mobile instant messaging–delivered psychoeducational intervention for cancer caregivers: a randomized clinical trial. *JAMA Netw Open*. 2024;7(2):e2356522. doi:10.1001/jamanetworkopen.2023.56522

eAppendix. Detailed Descriptions of Each Measurement

eTable 1. Baseline Characteristics Comparison Between Participants Who Completed the Study and Those Who Dropped Out of the Study

eTable 2. Generalized Estimating Equations Models for Different Dimensions of Quality of Life Across Time Between the Intervention and Control Groups

eReferences

This supplementary material has been provided by the authors to give readers additional information about their work.

© 2024 Cheng Q et al. *JAMA Network Open*.

eAppendix. Detailed descriptions of each measurement

Anxiety was measured using 7-item Generalised Anxiety Disorder scale, which is a self-reported scale consisting of 7 items that measure the respondents' experience of anxiety over the past two weeks. The response options range from 'not at all (0)' to 'nearly every day (3)'. The total score ranges from 0 to 21, with higher scores indicating higher levels of anxiety.[1,2] The Chinese version of the Generalized Anxiety Disorder scale demonstrates good internal consistency (Cronbach's alpha = 0.898) and test-retest reliability (ICC=0.856).[3] It also has good convergent validity with Hospital Anxiety and Depression Scale and Hamilton Depression Scale.[3] The GAD-7 has been widely used in caregivers of patients with cancer.[4-6]

Depression was measured by the Patient Health Questionnaire-9. It is an instrument used to measure the severity of depression in the general population during the past two weeks. A 4-point scale is used to rate the degree of severity from 'not at all (0)' to 'nearly every day (3)'. The total score of PHQ-9 ranges from 0 to 27, with higher scores indicating higher levels of depression.[7,8] The simplified Chinese version has shown good internal consistency (Cronbach's alpha = 0.857) and test-retest reliability (ICC= 0.857).[9] It also has good convergent validity with the hospital anxiety and depression scale and the Hamilton depression scale.[9] The scale is widely used in caregivers of patients with cancer to assess their depression level.[4-6]

Caregivers' quality of life was assessed using the Quality of Life Scale-Family Version.[10] The Chinese version has 35 items in total and four dimensions. Respondents rate each item on a linear scale ranging from 0 to 10. The total score ranges from 0 to 350, with higher scores implying better quality of life.[11] The Chinese version of the QOL-scale FAM has good reliability and validity, with a Cronbach' α of was 0.794 for the total questionnaire and the four factors accounting for 60.2% of the total variance. It has been used widely among caregivers of various patients with cancer.[12-14]

Caregivers' coping was evaluated by the Brief Coping Orientations to Problems Experienced Scale.[15,16] It comprises 28 items and can be categorised into three types of coping strategies: problem-focused strategies, emotion-focused strategies and dysfunctional coping strategies.[15] Each item is rated on a four-Likert scale ranging from 'I haven't been doing this at all (1)' to 'I've been doing this a lot (4)'. No overall score is available for the total scale. It advises adding the score of each subscale separately to determine the respondents' usage of each particular coping strategy. A higher score represents the greater utilisation of a specific coping strategy. The Chinese version of Brief-COPE has sound reliability,[16] and has been widely used among caregivers of with cancer to evaluate their coping methods.[17,18]

The Support Person's Unmet Needs Survey—Short Form was used to measure the caregivers' unmet needs.[19,20] The Chinese version is a 21-item validated scale used to measure the caregivers' unmet needs, including information needs, worries about future, financial needs, healthcare access and continuity, and personal and emotional needs.[20] All items are responded with a score of from 0 to 4, with scoring 3 or above classified as "High level" unmet needs.[19] Scores of the total score are summed up to indicate the unmet needs. A higher score represents a higher level of unmet need. It was validated in 1,026 Chinese caregivers of patients with cancer and showed good reliability and validity.[20] The internal consistency of the Chinese version of SPUNS-SF is high, with Cronbach's alpha coefficients of 0.94. The five factors extracted could account for 78.47% of the total variance, indicating a good construct.[20]

eTable 1. Baseline characteristics comparison between the participants who completed the study and those who dropped out of the study

Variable	Complete (N = 120) Mean ± SD/n (%)	Drop out (N = 40) Mean ± SD/n (%)	t/x^2	p-value
**Caregivers**				
Age, mean (SD), y	40.25 ± 8.09	40.33 ± 9.11	−0.049 [a]	.96
Sex			0.546 [b]	.46
Male	71 (59.2)	21 (52.5)		
Female	49 (40.8)	19 (47.5)		
Educational level			1.019	.80 [c]
Primary school or below	6 (5.0)	3 (7.5)		
Junior high school	37 (30.8)	10 (25.0)		
Senior high school	34 (28.3)	11 (27.5)		
College or above	43 (35.8)	16 (40.0)		
Marital status				.68 [c]
Married	114 (95.0)	39 (97.5)		
Unmarried, widowed,or divorced	6 (5.0)	1 (2.5)		
Place of residence			0.860 [b]	.35
Urban	47 (39.2)	19 (47.5)		
Rural	73 (60.8)	21 (52.5)		
Employment status			0.902 [b]	.34
Employed	79 (65.8)	23 (57.5)		
Not employed	41 (34.2)	17 (42.5)		
Monthly family income per capita, CNY			5.603	.23 [c]
≤1000	12 (10.0)	5 (12.5)		

© 2024 Cheng Q et al. *JAMA Network Open*.

Variable	Complete (N = 120) Mean ± SD/n (%)	Drop out (N = 40) Mean ± SD/n (%)	t/x^2	p-value
1001–3000	33 (27.5)	8 (20.0)		
3001–5000	32 (26.7)	12 (30.0)		
5001–8000	33 (27.5)	7 (17.5)		
≥8001	10 (8.3)	8 (20.0)		
Relationship to the patient			1.504	.49 [c]
Spouses/partner	79 (65.8)	25 (62.5)		
Parent	26 (21.7)	12 (30.0)		
Sibling	15 (12.5)	3 (7.5)		
**AYA patients with cancer**				
Age, mean (SD), y	33.28 ± 6.30	31.75 ± 7.46	1.271 [a]	.21
Sex			2.319 [b]	.13
Male	30 (25.0)	15 (37.5)		
Female	90 (75.0)	25 (62.5)		
Medical insurance			0.680 [c]	
No	7 (5.8)	1 (2.5)		
Yes	113 (94.2)	39 (97.5)		
Type of cancer			3.607	.75 [c]
Breast	37 (30.8)	12 (30.0)		
Gynacological	35 (29.2)	8 (20.0)		
Head and neck	13 (10.8)	7 (17.5)		
Lymphoma	10 (8.3)	5 (12.5)		
Gastrointestinal and liver	9 (7.5)	4 (10.0)		
Bone	9 (7.5)	3 (7.5)		
Other	7 (5.8)	1 (2.5)		

© 2024 Cheng Q et al. *JAMA Network Open*.

Variable	Complete (N = 120) Mean ± SD/n (%)	Drop out (N = 40) Mean ± SD/n (%)	t/x^2	p-value
Age when diagnosed, mean (SD), y	32.92 ± 6.39	31.18 ± 7.42	1.440 [a]	.15
Cancer stage			4.301	.37 [c]
I	26 (21.7)	6 (15.0)		
II	23 (19.2)	4 (10.0)		
III	36 (30.0)	14 (35.0)		
IV	26 (21.7)	10 (25.0)		
Unclear	9 (7.5)	6 (15.0)		
Current cancer treatment			0.093 [b]	.76
Surgery	33 (27.5)	12 (30.0)		
Chemotherapy	87 (72.5)	28 (70.0)		
**Outcome variables**				
Anxiety (GAD-7, Range: 0-21)	8.97 ± 5.55	10.40 ± 5.31	−1.429 [a]	.16
No (<5)	28 (23.3)	6 (15.0)	3.569 [b]	.31
Mild (5–9)	46 (38.3)	13 (32.5)		
Moderate (10–14)	21 (17.5)	12 (30.0)		
Severe (≥15)	25 (20.8)	9 (22.5)		
Depression (PHQ-9, Range: 0-27)	7.38 ± 6.00	8.75 ± 5.84	−1.256 [a]	.21
No (<5)	47 (39.2)	11 (27.5)	4.965	.29 [c]
Mild (5–9)	43 (35.8)	15 (37.5)		
Moderate (10–14)	17 (14.2)	6 (15.0)		
Moderately severe (15–19)	6 (5.0)	6 (15.0)		
Severe (≥20)	7 (5.8)	2 (5.0)		
Quality of life (QOL-scale FAM)				
Physical wellbeing (Range: 0-70)	43.26 ± 12.42	41.93 ± 9.18	0.724 [a]	.47

© 2024 Cheng Q et al. *JAMA Network Open*.

Variable	Complete (N = 120) Mean ± SD/n (%)	Drop out (N = 40) Mean ± SD/n (%)	t/x^2	p-value
Psychological wellbeing (Range: 0-100)	28.57 ± 19.59	32.83 ± 22.64	−1.144 [a]	.25
Social concerns (Range: 0-90)	47.73 ± 18.96	50.15 ± 18.32	−0.704 [a]	.48
Spiritual wellbeing (Range: 0-90)	53.63 ± 15.85	52.90 ± 16.61	0.250 [a]	.80
Overall quality of life (Range: 0-350)	173.19 ± 54.11	177.80 ± 53.83	−0.467 [a]	.64
Coping (Brief-COPE)				
Problem-focused coping (Range: 6-24)	15.35 ± 3.15	15.10 ± 3.12	0.436 [a]	.66
Emotion-focused coping (Range: 10-40)	22.01 ± 4.20	22.13 ± 4.04	−0.154 [a]	.88
Dysfunctional coping (Range: 12-48)	22.55 ± 4.46	24.18 ± 4.75	−1.964 [a]	.05
Unmet needs (SPUNS-SF)				
Information needs (Range: 0-24)	15.57 ± 6.17	13.93 ± 5.67	1.486 [a]	.14
Worries about future (Range: 0-12)	8.97 ± 3.10	8.50 ± 3.44	0.802 [a]	.42
Financial needs (Range: 0-8)	3.98 ± 2.59	3.63 ± 2.55	0.742 [a]	.46
Healthcare access and continuity (Range: 0-20)	11.98 ± 5.92	11.75 ± 6.15	0.206 [a]	.84
Personal and emotional needs (Range: 0-20)	10.07 ± 5.08	9.05 ± 4.84	1.119 [a]	.27
Total unmet needs (Range: 0-84)	50.56 ± 17.88	46.85 ± 16.47	1.158 [a]	.25

Note. t = Independent t-tests; x^2 = Chi-square tests; CNY = Chinese Yuan.GAD-7 = Seven-item Generalized Anxiety Disorder scale; PHQ-9 = Patient Health Questionnaire-9; QOL-scale FAM = Quality of Life Scale-Family Version; Brief-COPE = Brief Coping Orientations to Problems Experienced Scale; SPUNS-SF = Support Person’s Unmet Needs Survey—Short Form.

[a] Independent t-tests were performed. [b] Chi-square tests were performed. [c] Fisher’s exact test.

© 2024 Cheng Q et al. *JAMA Network Open*.

eTable 2. Generalized estimating equations models for different dimensions of quality of life across time between the intervention and control groups

Outcome variables	Mean (SD)	Mean (SD)	Group coefficient		Time coefficients		Group * Time coefficients		Cohen ’ d
	Intervention	Control	B (95% CI)	*p*	B (95% CI)	*p*	B (95% CI)	*p*	
Quality of life (QOL-scale FAM, Range: 0-350)									
Physical wellbeing (Range: 0-70)									
T0	43.65 ± 13.08	42.20 ± 10.12	1.450 (−2.152, 5.052)	.43	NA	NA	NA	NA	NA
T1	40.59 ± 12.42	37.61 ± 12.50	NA		−4.895 (−7.527, −2.264)	<.001	2.163 (−1.463, 5.788)	.24	NA
T2	38.80 ± 13.87	38.02 ± 12.04	NA		−4.770 (−7.174, −2.365)	<.001	−0.262 (−4.016, 3.492)	.89	NA
Psychological wellbeing (Range: 0-100)									
T0	30.09 ± 21.46	29.18 ± 19.41	0.912 (−5.389, 7.214)	.78	NA	NA	NA	NA	NA
T1	33.13 ± 22.52	28.20 ± 17.75	NA		−1.785 (−5.342, 1.773)	.33	5.230 (0.277, 10.184)	.04	0.34
T2	33.53 ± 19.57	28.95 ± 19.25	NA		−0.400 (−3.761, 2.960)	.82	4.379 (−0.631, 9.390)	.09	NA
Social concerns (Range: 0-90)									
T0	49.56 ± 20.17	47.11 ± 17.30	2.450 (−3.336, 8.236)	.41	NA	NA	NA	NA	NA
T1	47.30 ± 16.99	39.99 ± 15.49	NA		−7.984 (−11.345, −4.623)	<.001	5.914 (1.053, 10.774)	.02	0.38

© 2024 Cheng Q et al. *JAMA Network Open*.

Outcome variables	Mean (SD)	Mean (SD)	Group coefficient		Time coefficients		Group * Time coefficients		Cohen' d
	Intervention	Control	B (95% CI)	*p*	B (95% CI)	*p*	B (95% CI)	*p*	
T2	44.55 ± 19.13	40.82 ± 16.27	NA		−6.836 (−10.109, −3.564)	<.001	2.174 (−2.615, 6.963)	.37	NA
Spiritual wellbeing (Range: 0-90)									
T0	55.35 ± 17.83	51.55 ± 13.77	3.800 (−1.105, 8.705)	.13	NA	NA	NA	NA	NA
T1	53.06 ± 15.88	49.09 ± 13.45	NA		−2.316 (−5.470, 0.837)	.15	0.119 (−4.685, 4.922)	.96	NA
T2	51.83 ± 15.85	48.53 ± 13.44	NA		−3.134 (−6.097, −0.171)	.04	−0.373 (−5.115, 4.370)	.88	NA

Note. T0 = Baseline; T1 = 5 weeks after baseline; T2 = 12 weeks after baseline; B = Regression coefficients of the dummy variables for group [Group: 0= Control (reference); 1= intervention], time points (T1 and T2 with the baseline (T0) as reference), time points and group interaction terms (Group*Time); CI = Confidence interval; QOL-scale FAM = Quality of Life Scale-Family Version; NA = Not applicable.

© 2024 Cheng Q et al. *JAMA Network Open*.

eReferences

1. Lowe B, Decker O, Muller S, et al. Validation and standardization of the Generalized Anxiety Disorder Screener (GAD-7) in the general population. *Medical Care.* 2008;46(3):266–274.doi: 10.1097/MLR.0b013e318160d093
2. Spitzer RL, Kroenke K, Williams JB, Lowe B. A brief measure for assessing generalized anxiety disorder: the GAD-7. *Archives of Internal Medicine.* 2006;166(10):1092–1097.doi: 10.1001/archinte.166.10.1092
3. He X, Li C, Qian J, Cui H, Wu W. Reliability and validity of a generalized anxiety disorder scale in general hospital outpatients. *Shanghai Archives of Psychiatry.* 2010;22(4):200–203.doi.
4. Parker Oliver D, Washington K, Smith J, Uraizee A, Demiris G. The prevalence and risks for depression and anxiety in hospice caregivers. *Journal of Palliative Medicine.* 2017;20(4):366–371.doi: 10.1089/jpm.2016.0372
5. Ullrich A, Ascherfeld L, Marx G, Bokemeyer C, Bergelt C, Oechsle K. Quality of life, psychological burden, needs, and satisfaction during specialized inpatient palliative care in family caregivers of advanced cancer patients. *BMC Palliative Care.* 2017;16(1):31.doi: 10.1186/s12904-017-0206-z
6. Oechsle K, Ullrich A, Marx G, et al. Psychological burden in family caregivers of patients with advanced cancer at initiation of specialist inpatient palliative care. *BMC Palliative Care.* 2019;18(1):102.doi: 10.1186/s12904-019-0469-7
7. Martin A, Rief W, Klaiberg A, Braehler E. Validity of the Brief Patient Health Questionnaire Mood Scale (PHQ-9) in the general population. *General Hospital Psychiatry.* 2006;28(1):71–77.doi: 10.1016/j.genhosppsych.2005.07.003
8. Kroenke K, Spitzer RL, Williams JB. The PHQ-9: Validity of a brief depression severity measure. *Journal of General Internal Medicine.* 2001;16(9):606–613.doi: 10.1046/j.1525-1497.2001.016009606.x
9. Bian C, He X, Qian J, Wu W, Li C. The reliability and validity of a modified patient health questionnaire for screening depressive syndrome in general hospital outpatients. *Journal of Tongji University (Medical Science).* 2009;30(5):136-140.doi.
10. Ferrell BR, Grant M. Quality of Life Family Version. https://www.cityofhope.org/doc/1431763601545-qol-family.pdf. Published 2005. Accessed.
11. Liu Y, Gan X, Tang W, Li X. Application of QOL Scale-FAMILY in Evaluation of Quality of Life for Caregivers of Patients with Laryngocarcinoma. *Chinese General Practice.* 2009;12(5A):726-728.doi: 1007-9572(2009) 05-0726-03
12. Jin M, Li L, Yao J. Role of social support in the quality of life and burden of care of primary caregivers of patients with liver cancer. *Nursing Journal of Chinese People's Liberation Army.* 2020;37(05):65-68+75.doi.
13. Luan B, Wang W, Zhu Y. The relationship between the care burden and quality of life among caregivers of elderly cancer patients receiving chemotherapy. *Chinese Journal of Gerontology.* 2017;37(20):5162-5164.doi.
14. Wu L, Gao J, Gu J, Yu Y, Jiang L. Effect of family intervention on caregiver burden and quality of life of lung cancer patients. *Shanghai Nursing.* 2020;20(09).doi.
15. Carver CS. You want to measure coping but your protocol's too long: Consider the brief COPE. *International Journal of Behavioral Medicine.* 1997;4(1):92–100.doi: 10.1207/s15327558ijbm0401_6
16. Wang XQ, Lambert CE, Lambert VA. Anxiety, depression and coping strategies in post-hysterectomy Chinese women prior to discharge. *International Nursing Review.* 2007;54(3):271–279.doi: 10.1111/j.1466-7657.2007.00562.x
17. Han Y, Hu D, Liu Y, et al. Coping styles and social support among depressed Chinese family caregivers of patients with esophageal cancer. *European Journal of Oncology Nursing.* 2014;18(6):571–577.doi: 10.1016/j.ejon.2014.07.002
18. Wang T, Molassiotis A, Tan JY, Chung BPM, Huang HQ. Prevalence and correlates of unmet palliative care needs in dyads of Chinese patients with advanced cancer and their informal caregivers: a cross-sectional survey. *Supportive Care in Cancer.* 2021;29(3):1683–1698.doi: 10.1007/s00520-020-05657-w
19. Campbell SH, Carey M, Sanson-Fisher R, et al. Measuring the unmet supportive care needs of cancer support persons: the development of the support person's unmet needs survey--short form. *European Journal of Cancer Care.* 2014;23(2):255–262.doi: 10.1111/ecc.12138
20. Han Y, Zhou Y, Wang J, et al. Chinese version of the Cancer Support Person's Unmet Needs Survey-Short Form: A psychometric study. *European Journal of Cancer Care.* 2019;28(2):e12963.doi: 10.1111/ecc.12963

© 2024 Cheng Q et al. *JAMA Network Open.*
