# Supplementary material for: A Mobile Instant Messaging–Delivered Psychoeducational Intervention for Cancer Caregivers: A Randomized Clinical Trial
Source: JAMA Netw Open. 2024 Feb 22;7(2):e2356522. doi: 10.1001/jamanetworkopen.2023.56522 (PMC10884881; doi:10.1001/jamanetworkopen.2023.56522)
Supplement: Supplement 3. — Data Sharing Statement [file jamanetwopen-e2356522-s003.pdf]

Data Sharing Statement

Cheng. A Mobile Instant Messaging–Delivered Psychoeducational Intervention for Cancer Caregivers. *JAMA Netw Open*. Published February 22, 2024.
doi:10.1001/jamanetworkopen.2023.56522

Data
Data available: Yes
Data types: Deidentified participant data
How to access data: The data used in this study are available from the corresponding author on a reasonable request. Email address: winnieso@cuhk.edu.hk
When available: With publication

Supporting Documents
Document types: None

Additional Information
Who can access the data: Researchers whose proposed use of the data has been approved
Types of analyses: For a specified purpose
Mechanisms of data availability: After approval of a proposal
Any additional restrictions: N/A
